# Direct Bactericidal Comparison of Metal Nanoparticles and Their Salts against *S*. *aureus* Culture by TEM and FT-IR Spectroscopy

**DOI:** 10.3390/nano12213857

**Published:** 2022-11-01

**Authors:** Irina Saraeva, Eteri Tolordava, Yulia Yushina, Islam Sozaev, Vera Sokolova, Roman Khmelnitskiy, Svetlana Sheligyna, Tatiana Pallaeva, Nikolay Pokryshkin, Dmitry Khmelenin, Andrey Ionin, Anastasia Semenova, Sergey Kudryashov

**Affiliations:** 1 P. N. Lebedev Physics Institute, Russian Academy of Sciences, 119991 Moscow, Russia; 2 V. M. Gorbatov Federal Research Center for Food Systems, Russian Academy of Sciences, 109316 Moscow, Russia; 3 N. F. Gamaleya Federal Research Centre of Epidemiology and Microbiology, 123098 Moscow, Russia; 4 Institute of Crystallography, Branch of the Federal Scientific Research Centre “Crystallography and Photonics”, Russian Academy of Sciences, 119333 Moscow, Russia; 5 Faculty of Physics, M. V. Lomonosov Moscow State University, 119991 Moscow, Russia

**Keywords:** Fourier-transform IR spectroscopy, silver, nanoparticles, antibacterial properties

## Abstract

We report the bactericidal effect of Ag and Cu NPs with different concentrations on methicillin-resistant *S. aureus* strain in comparison to the effect of AgNO_3_ and CuCl_2_ solutions, characterized by microbiological tests, TEM and Fourier-transform infrared spectroscopy. NPs were produced by nanosecond laser ablation in distilled water and characterized by scanning electron microscopy, UV-vis, energy dispersive X-ray, FT-IR spectroscopy, as well as X-ray diffraction, dynamic light scattering size and zeta-potential measurements. Microbiological tests showed antibacterial activity of NPs and metal ion-containing salts. Comparative FT-IR spectroscopy of bacteria, treated with metal NPs and salts, showed the broadening of amide I and II bands, a CH_2_-related peak and its frequency decrease, indicating the increase of membrane fluidity. The main mechanisms of the antibacterial effect were proposed: Ag and Cu NPs release ions and ROS, which result in lipid peroxidation; AgNO_3_ forms precipitates on the cell surface, which lead to the mechanical rupture of the membrane and subsequent possible penetration of the precipitates in the emerged damaged spots, complete destruction of the membrane and bacterial death; Cu ions from the CuCl_2_ solution cause damage to phosphorus- and sulfur-containing biomolecules, which leads to disruption of intracellular biochemical processes. The theories were confirmed by FT-IR spectroscopy and TEM.

## 1. Introduction

In order to enhance the growth of livestock for further meat processing, antibiotics are often added to the nutrition in small doses over long periods of time, thus preventing possible diseases and death [1,2]. Needless to say, such a deliberate waste of antibiotics has a highly negative impact on human health [3], causing dysbacteriosis and allergic reactions. Pathogenic bacteria, being in the long-term presence of antibiotics, start to adjust by gene mutations, acquiring strong resistivity to commonly used drugs [4,5]. The specter of drugs, to which bacteria have developed resistivity, is growing, and we are in need of alternative methods for antibacterial procedures.

Such methods include the use of nanostructures and nanoparticles, which are affective either by direct mechanical damage to the cell membrane [6,7], or act through the formation of reactive oxygen species (ROS) [8,9]. Several theories have been proposed, such as the reaction of these metals with proteins by combining the -SH groups of enzymes, leading to the inactivation of the proteins [10]. A recent article [11] has proposed the optimal conditions for strong antibacterial effect of Ag NPs—their surface chemistry has to be controlled in order to elude aggregation and produce Ag^+^ ions, and its charge has to be positive to enhance the binding with bacterial cell membrane, while NP size should be 10 nm or less. Silver and copper NPs are widely known for their bactericidal action on microorganisms both in planktonic form and as durable biofilms [10,11,12,13,14,15,16]. Twelve-nm Cu NPs, prepared by inert gas condensation method, were effectively used against *E. coli* [13]. It was observed that Cu NPs damage cells by leaving cavities in the bacterial cell wall. Moreover, silver and copper ions can be used as disinfectants for wastewater from hospitals [17,18]. Copper NPs have proved to be more effective in antibacterial therapy than silver on *E. coli* and *Bacillus subtilis* [19].

Unlike metal NPs [20,21], ion-containing solutions are used more rarely in antimicrobial applications. The formation of dense granules around the AgNO_3_-treated bacteria, consisting of silver and sulfur, has been previously discovered to occur [22]. The comparison of the inhibition zone diameter after treatment of pathogenic bacteria with Ag NPs and AgNO_3_ was also carried out, and it was found out that, for NPs, the diameters were two-times higher [23]. Cu ion-containing solutions have also been previously tested against several microorganisms. It has been suggested, that, at given concentrations, Cu interacts with cellular nucleic acids and enzyme active sites, as well as plasma membrane [24,25,26,27,28,29], and that treatment of microorganisms with Cu can interfere with membrane integrity, which is accompanied by leakage of mobile cellular solutes such as K^+^ and the subsequent cell death [25]. A two-fold increase of cellular K^+^ release was observed after addition of Cu(NO_3_)_2_ to *Saccharomyces cerevisiae*—a yeast used in brewery and baking [27].

AgNO_3_ has been found to exhibit antimicrobial activity with a minimal inhibitory concentration of three μM against *E. coli* [30]. The bactericidal effect of Ag nanoparticles at millimolar levels (2–10 mM) has been previously studied on mesophilic and halophilic bacteria spp. such as *Enterobacter*, *Bacillus subtilis* and *Marinobacter* [31]. Bactericidal activity of Ag NPs has been detected at low concentrations against many bacterial strains. Guzman et al. reported bactericidal activity of Ag NPs against *E. coli*, *S. aureus* and *Pseudomonas aeruginosa* at ∼216 ppm (parts per million) [32]. Matzke et al. found that Ag NPs inhibited *Pseudomonas putida* growth at 3.4 g/L concentration [33].

AgNO_3_ can also be used as a catalyst for the preparation of biocompatible NPs, which may then be loaded with antimicrobial agents. The synthesis of low molecular weight chitosan NPs by ionotropic gelation of depolymerized chitosan for effective drug delivery in drug resistant bacteria in the presence of silver nitrate has been reported [34]. The resulting tetracycline-loaded NPs were then successfully used for antibacterial treatment on the strain of tetracycline-resistant *E. coli*. The authors also used AgNO_3_ for synthesis of low molecular weight alginic acid NPs for effective drug delivery to drug resistant bacteria [35].

Thus, Ag- and Cu-containing agents are effective against a plethora of microorganisms, but in order to define optimal treatment one has to include such methods of characterization as FT-IR spectroscopy, which allows the elucidation of the main differences in molecular structure of bacterial cells before and after NP treatment. Several studies address the issue of bacterial prototyping for subsequent database formation [36,37,38,39,40,41]. Some articles report the changes in FT-IR spectra and conclude that it is a promising method for the control and study of antibacterial agents.

The main changes in bacterial cell structure lead to the variation of width, intensity and area of bands, corresponding to specific chemical compounds, with the Lorentzian fit of the peaks giving the necessary information. The overall disruption of the membrane leads to a change in the lipid bilayer—the so-called increase of membrane fluidity [42]. The bands at 2854 cm^−1^ and 2924 cm^−1^ correspond to symmetric and asymmetric stretching vibrations of CH_2_, respectively, and their broadening indicates the molecular disorder in the membrane, which may be caused by metal ion adhesion to bacterial cells [43,44]. Amide I and II bandwidths illustrate conformational freedom of proteins [43,44], and their narrowing indicate an increase in protein binding, leading to the flexibility decrease caused by the proteins’ binding to metal ions.

TEM analysis is also a necessary tool for correct understanding of bacterial death mechanisms. It allows the detection of the penetration of NPs through the cell membrane and the subsequent damage [45].

In our work, we report the comparative analysis of Ag and Cu antibacterial effect in the form of NP colloidal solutions and ion-containing chemical compounds (AgNO_3_, CuCl) on *S. aureus* strain, supported with FT-IR spectroscopy data, TEM and SEM characterization along with EDS analysis and subsequent microbiological tests, confirming antibacterial effect.

## 2. Materials and Methods

For the comparison of the antibacterial effect of Ag-/Cu NPs and Ag-/Cu ion-containing salts we chose AgNO_3_ and CuCl_2_. Solutions with several concentrations were prepared for the subsequent bactericidal treatment: 10.8 mM, 7.2 mM and 3.6 mM AgNO_3_ solutions of metal-ion containing salts and Ag NPs colloid, fabricated in distilled water and then diluted resulting in 0.3 mg/mL, 0.18 mg/mL and 0.07 mg/mL concentration. For experiments with Cu, CuCl_2_ solutions were prepared with 51.6 mM, 12.4 mM and 6.2 mM concentrations, and Cu NPs colloids were diluted analogically to Ag NPs (0.3 mg/mL, 0.18 mg/mL and 0.07 mg/mL concentrations).

Colloidal Ag and Cu NPs were obtained via nanosecond laser ablation (laser system HTF-Mark, Bulat, wavelength λ = 1030 nm, pulsewidth τ = 120 ns) of 3 × 3 mm^2^ area on a bulk Ag (Sber, Moscow, Russia, 99.999% purity) or Cu target (P. N. Lebedev Physics Institute, Moscow, Russia, 99.999% purity) under the distilled water layer (thickness h ≈ 2 mm). Laser radiation was focused with an F-theta objective (focal length F = 160 mm) into a spot with 1/e diameter d ≈ 20 µm and raster-scanned across the area with the use of galvanometric scanning head (Ateko) with constant speed v = 60 mm/s, pulse repetition rate f = 20 kHz, average pulse energy E = 0.6 mJ, and for N = 40 times. As a result, the liquid above the target acquired either greyish-yellow color, which indicated the formation of Ag nanoparticles, or dark green in case of Cu NPs.

Surface topography and chemical composition of the samples were characterized by scanning electron microscope (SEM) TESCAN Vega (Tescan JSCo, Brno, Czech Republic), equipped with an energy-dispersive X-ray spectroscopy (EDS) module for the elemental composition analysis, by transmission electron microscope (TEM) Tecnai Osiris (FEI company, Hillsboro, OR) operating at 200 keV and equipped with Super-X SDD high-sensitivity EDS module. For SEM imaging a 5-µL drop of each sample was placed on a Si substrate and dried at room temperature. For TEM analysis the similar amount of liquid solution was placed on a carbon-coated golden grid.

XRD spectra were obtained with DR-02 RADIAN diffractometer (JSC Expertcentr, Moscow, Russia), using Cu Kα X-rays with wavelength λ = 0.154 nm. Data were recorded for the 2*θ* range of 10–60° with a 0.05 step.

For FT-IR spectral characterization, a drop of each sample (10 μL) was placed on a Si substrate and dried. Measurements were acquired in vacuum camera with the use of 2-mm diaphragm, in the range 800–3500 cm^−1^, using spectrometer V-70 (Bruker). Pure fluorite substrate and samples with deposited Ag and Cu oxide NPs were also measured for subsequent spectral normalization. After measurement of IR transmittance, all spectra were converted to optical density and normalized, after which their comparative analysis was implemented.

UV-vis spectra of all solutions were measured with SF-2000 spectrometer (190–1100 nm measurement range, resolution 0.1 nm), and NP size distributions and z-potential were measured by Zeta Sizer spectrometer (Malvern Instruments, Inc., Malvern, UK), with each value being averaged from three parallel measurements.

For biofilm preparation, substrates with areas 1 × 1 cm^2^ were cut from a large silicon (Si) plate (thickness 380 µM, purity 99.99%) for subsequent samples analysis. Bacterial isolates were provided by V. M. Gorbatov Federal Research Center for Food Systems of Russian Academy of Sciences. The over-night (18-h) culture was centrifuged with the subsequent removal of supernatant. The sedimented bacterial cells were intensively shaken with distilled water, after what the resulting suspension was then subjected to serial logarithmic dilutions up to 10^5^ CFU/mL (colony-forming unit per milliliter).

For bactericidal treatment, biofilms of *S. aureus* were grown for 24 h on Si substrates. Several samples were prepared: untreated *S. aureus* biofilm for control, and biofilms treated with AgNO_3_, Ag NPs, CuCl_2_ and Cu NPs solutions with different concentrations, three of each. Drops of each solution (10 µL) were put on grown biofilms and left for 20 min, during which the complete evaporation of liquid occurred.

Each sample was also duplicated for parallel inoculation. One mL was taken from the biofilms in order to determine the number of CFU in the original sample (control). All samples were then titrated, plated on Petri dishes with solid nutrient medium and left for 24-h incubation, after which the number of CFU was determined.

## 3. Results

### 3.1. Characterization of NP Colloids and Solutions

**Figure 1:**
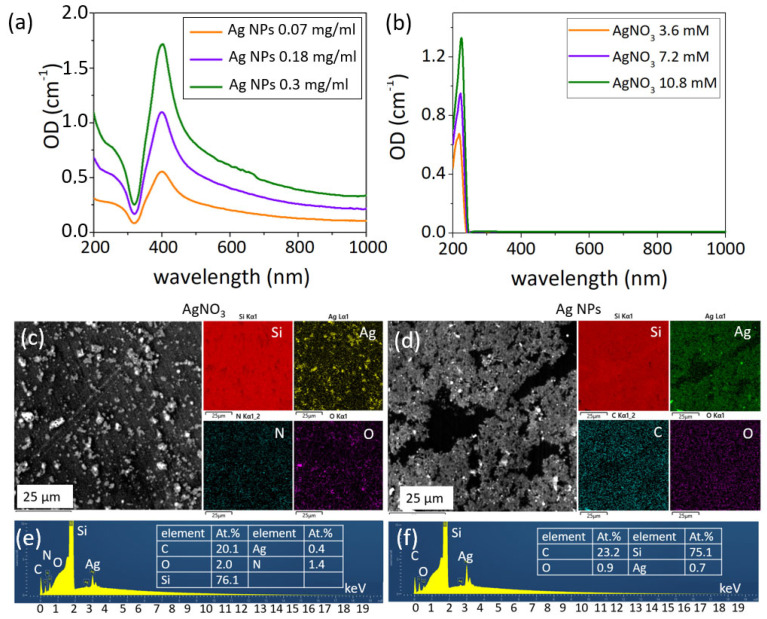
UV−vis spectra of (**a**) Ag NP colloids and (**b**) AgNO_3_ solutions with different concentrations; SEM and EDS characterization of AgNO_3_ solution (**c**,**e**) and Ag NP colloid (**d**,**f**), dried on a Si substrate. Insets on (**e**,**f**): tables of elements.

Ag NPs colloids have an absorption band at ≈400 nm (Figure 1a), corresponding to the localized plasmon resonance [46]. Absorption of the colloids diminishes with lower concentrations. AgNO_3_ solution has a weak absorption band at ≈300 nm (Figure 1b), which is in agreement with the literature [47] and may be attributed to interband transitions in silver (4d → 5s, p) [48].

SEM characterization showed that AgNO_3_ solution, dried at silicon wafers, forms large precipitates (Figure 1c). Ag NPs have a round shape and a moderate polydispersity in size distribution (Figure 1d). Results of EDS, obtained at accelerating voltage of 10 keV, show the high content of oxygen in dried AgNO_3_ drop (1 to 5 ratio of silver to oxygen) (Figure 1e) and its significantly lower value for Ag NPs (Ag—0.7 at.%, O—0.9 at.%) (Figure 1f), which therefore prove the moderate oxidation of waterborne Ag NPs.

**Figure 2:**
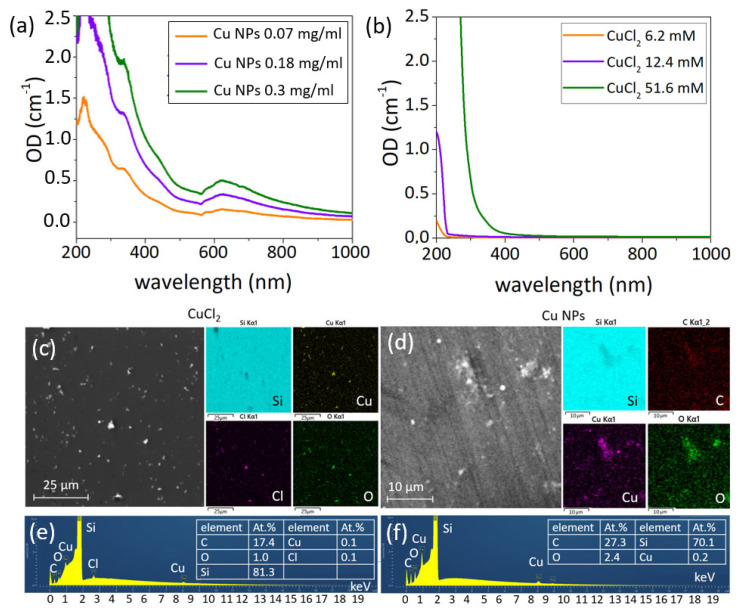
UV−vis spectra of (**a**) Cu NP colloid and (**b**) CuCl_2_ solutions with different concentrations. SEM and EDS characterization of CuCl_2_ solution (**c**,**e**) and Cu NP colloid (**d**,**f**) dried on a Si substrate. Insets on (**e**,**f**): tables of elements.

Cu NPs in colloidal form have a weak absorption band at ≈600 nm (Figure 2a), which corresponds to previously obtained data in the literature [49]. CuCl_2_ solutions have an absorption band at ≈200–250 nm (Figure 2b), which is in agreement with the literature [50] and disappears with a lowering of the solution concentration.

SEM characterization showed the formation of precipitates during the evaporation of liquid and drying of CuCl_2_, which consist of Cu, Cl and O (Figure 2c). Both these precipitates and waterborne Cu NPs (Figure 2d) exhibit strong oxidation—0.1 at.% (Cu) to 1 at.% (O) for CuCl_2_ (Figure 2e), and 0.2 at.% (Cu) to 2.4 at.% (O) for Cu NPs (Figure 2f).

**Figure 3:**
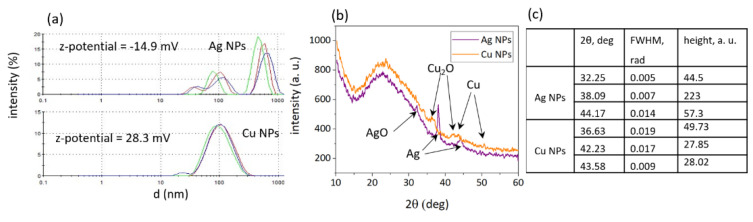
(**a**) DLS size distribution of Ag and Cu NPs with corresponding Z-potential values (green, red and purple lines represent the results of three measurements of the same samples); (**b**) XRD spectra of Ag (purple line) and Cu (orange line) NPs; (**c**) data of XRD pattern of Ag and Cu NPs.

Ag NPs, fabricated by laser ablation of bulk target, have three observable fractions with mean sizes of ≈38, 100 and 590 nm (Figure 3a) and negative z-potential −14.9 mV, whereas Cu NPs have main peaks at ≈25, 117 nm and a positive z-potential 28.3 mV (Figure 3a).

XRD spectra (Figure 3b) illustrate the phase composition of NPs, and have several diffraction peaks, which indicate the crystalline structure of both types of NPs. For the spectra registration, NPs from the colloidal solution were dried on a cover glass slide, therefore resulting in a broad fluorescence band from 2*θ* ~15 to 35° (Figure 3b). The observed diffraction peaks at angle 2*θ* ∼ 36.5°, 42° and 50.6° with (111), (002), and (200) planes correspond to face centered cubic structure (*fcc*) of Cu NPs [51]. Peaks at 2*θ* ∼ 32.2°, 38° and 44.2° with (220), (111), and (200) planes correspond to *hcp* structured nanoparticles [52]. The presence of both *fcc* and *hcp* structures is attributed to the growth along different crystallographic planes during the formation of NPs [53]. Cu NPs are presented in form of Cu_2_O and Ag NPs—AgO (Figure 3b). The crystallite size may be defined from the obtained spectra with the use of Scherer Formula (1):

(1) D_h,k,l_ = 0.9 *λ*/(*β*_h,k,l_cos*θ*)

where *λ* = 0.154 nm (Cu Kα-line) is the X-ray wavelength, *β* is the full linewidth at half maximum (FWHM), and *θ* is the diffraction angle at the corresponding planes.

In order to get values of FWHM a Lorentz fit was implemented for each peak in Origin software, resulting in data (Figure 3c) that show the average size of Ag NP crystallites equals to ≈ 20 nm, and for Cu NP crystallites ≈11 nm, possibly illustrating the several-nm fraction in DLS size distributions (Figure 3a).

### 3.2. TEM Characterization of NP-Treated Cells

**Figure 4:**
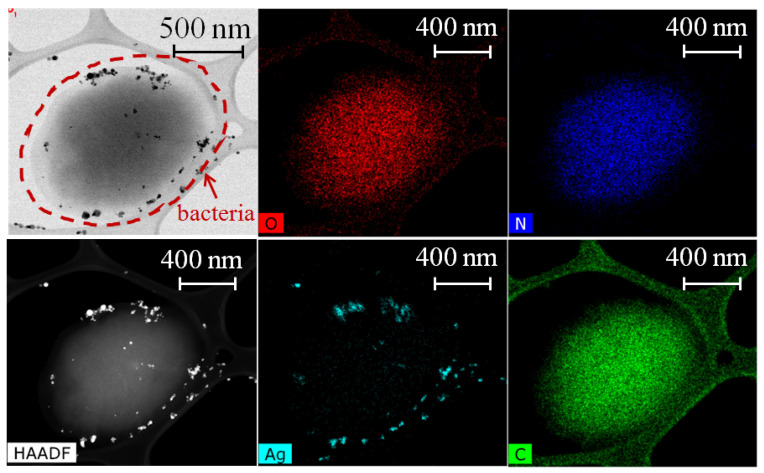
TEM image of *S. aureus* cells after treatment with AgNO_3_ 10.8 mM-solution, distribution of elements in the sample, acquired by EDS and HAADF image.

**Figure 5:**
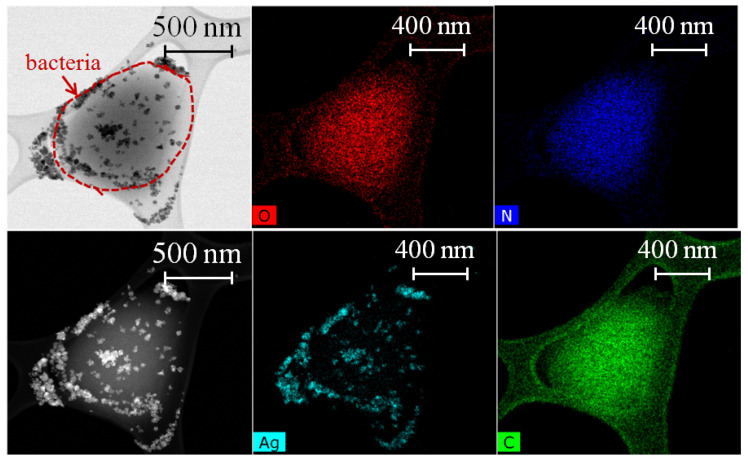
TEM image of *S. aureus* cells after treatment with Ag NPs colloid (concentration 1 mg/mL), distribution of elements in the sample, acquired by EDS and HAADF image.

For bactericidal treatment, biofilms of *S. aureus* were grown for 24 h on Si substrates. Several samples were prepared: untreated *S. aureus* biofilm for control, and biofilms treated with AgNO_3_, Ag NPs, CuCl_2_ and Cu NPs solutions with different concentrations, three of each. Drops of each solution (10 μL) were put on grown biofilms and left for 20 min, during which the complete evaporation of liquid occurred. TEM images of *S. aureus* cells, treated with AgNO_3_ and Ag NPs (see Section 2 for details), along with a high-angle annular dark-field (HAADF) image, are shown in Figure 4 and Figure 5. According to EDS analysis, silver nitrate solution has precipitated in the form of nanoparticles on the cell surface, which may have ruptured the membrane and contributed to its bactericidal effect. The precipitate may have also penetrated the cell membrane, as the mean size of particles equals ≈ 8–10 nm (Figure 5). Despite its content in AgNO_3_ solution, N content was only observed in the volume of bacteria (Figure 4 and Figure 5), presumably from the glycyl amide nitrogens in peptidoglycan and the esterified D-alanyl amine nitrogens in the wall teichoic acid [54].

**Figure 6:**
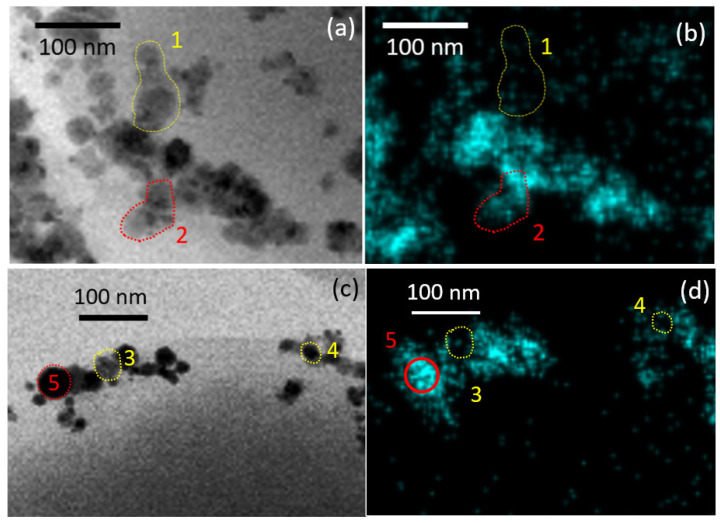
Enlarged TEM images of *S. aureus* cell after treatment with (**a**) Ag NPs solution; (**c**) treated with AgNO_3_and; and (**b**,**d**) the corresponding EDS map of Ag content in (**a**,**c**). Yellow shapes highlight NPs, visible on TEM images but absent in EDS maps. Red shapes highlight NPs visible both in TEM image and EDS maps. See text for details.

One can suggest the possible penetration of Ag NPs and Ag precipitates in the cell membrane by comparison of TEM image and the corresponding EDS of Ag distribution in the cell volume (Figure 6). The marked yellow (1) and red (2) shapes of Ag NPs on TEM image (Figure 6a) have the same contrast, which suggests the equal penetration depth of the electron beam, considering the exact same elemental composition of bacterial cell and NPs. The corresponding EDS map of the same area (Figure 6b) shows that, while shape 2 (red) is detected, shape 1 (yellow) is not seen. This may be explained by the longer path of the electron beam not reaching NPs inside the cell and therefore blocking the emission of characteristic X-rays. It is known that SEM EDS analysis of bulk samples obtain data from a pear-shaped interaction volume [55,56], thus acquiring elemental composition at high values of accelerating voltage (about 20 keV) not only from the surface and subsurface layers, but also (mainly) from the bulk of the material. TEM EDS analysis, on the other hand, deals with samples with thicknesses ≈ 80 nm, where the interaction volume is limited by the aperture size of the electron beam [57]. The minimization of interaction volume provides better spatial resolution [58], therefore the observed differences in EDS maps for Ag NP-treated *S. aureus* cells cannot be attributed to artefacts.

Likewise, Ag precipitate in area 3 (yellow) in Figure 6c,d is not seen in the corresponding EDS map. Areas 4 (yellow) and 5 (red) also have the same contrast, although while area 5 is clearly seen in the EDS map, area 4 is absent. This comparison also suggests the penetration of precipitates in the volume of bacterial cell.

### 3.3. FT-IR Spectral Measurements

**Figure 7:**
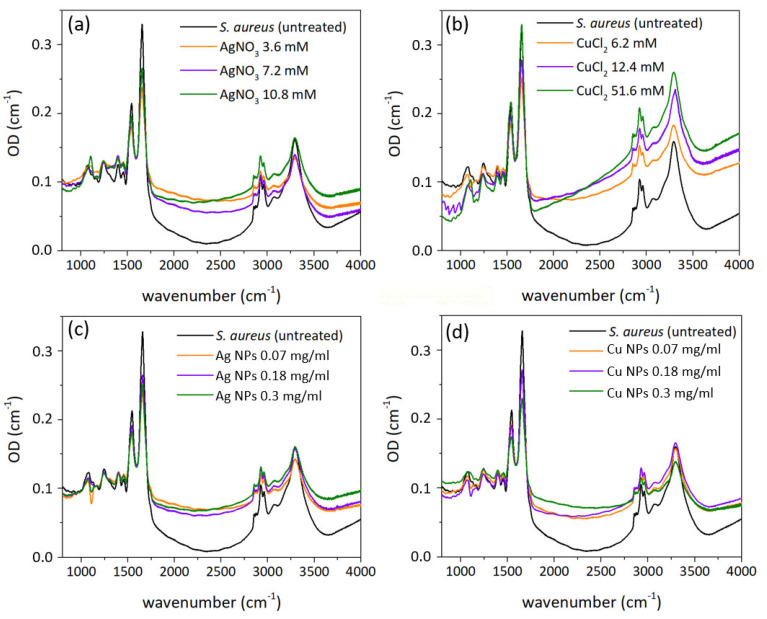
FT−IR optical density (OD) spectra of *S. aureus* before and after treatment with (**a**) AgNO_3_, (**b**) CuCl_2_, (**c**) Ag NPs, and (**d**) Cu NPs solutions with different concentrations.

For bactericidal treatment, biofilms of *S. aureus* were grown for 24 h on Si substrates. Several samples were prepared: untreated *S. aureus* biofilm for control, and biofilms treated with AgNO_3_, Ag NPs, CuCl_2_ and Cu NPs solutions with different concentrations, three of each. Drops of each solution (10 μl) were put on grown biofilms and left for 20 min, during which the complete evaporation of liquid occurred. FT-IR spectra of all prepared samples are presented in Figure 7 (see Section 2 for detailed description of FT-IR measurements). Optical density values were calculated from the initial data by formula OD = lg(T_substrate_/T).

Gram-positive bacteria have a thick layer of peptidoglycan (PG, 40–80% of the cell wall) [59]. Their primary structure consists of parallel polysaccharide chains of alternating N-acetylglucosamine (NAG) and N-acetylmuramic acid (NAM) residues joined by β(1 → 4) glycosidic bonds with parallel chains being linked by penta- or tetrapeptides. Gram-positive cell walls also contain teichoic acids that are covalently bound to the PG.

**Figure 8:**
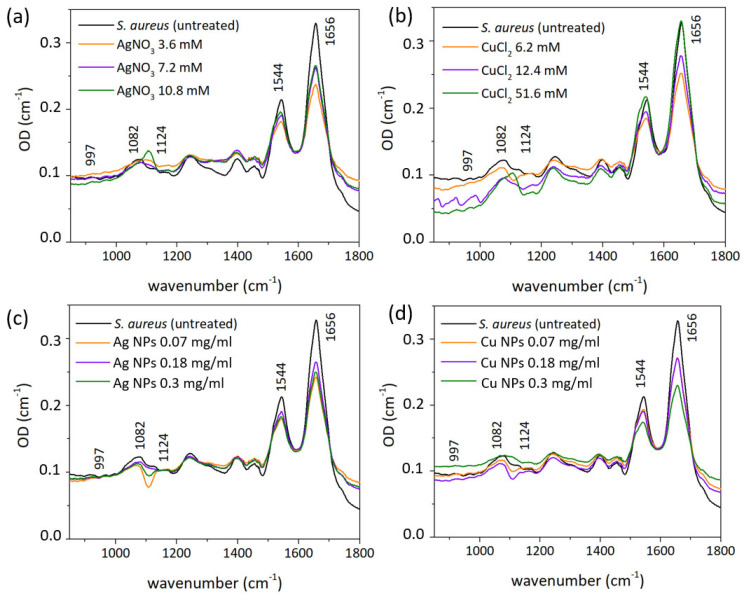
Selected regions (850–1800 cm^−1^) of FT−IR spectra of *S. aureus* before and after treatment with (**a**) AgNO_3_, (**b**) CuCl_2_, (**c**) Ag NPs, and (**d**) Cu NPs solutions with different concentrations.

**Figure 9:**
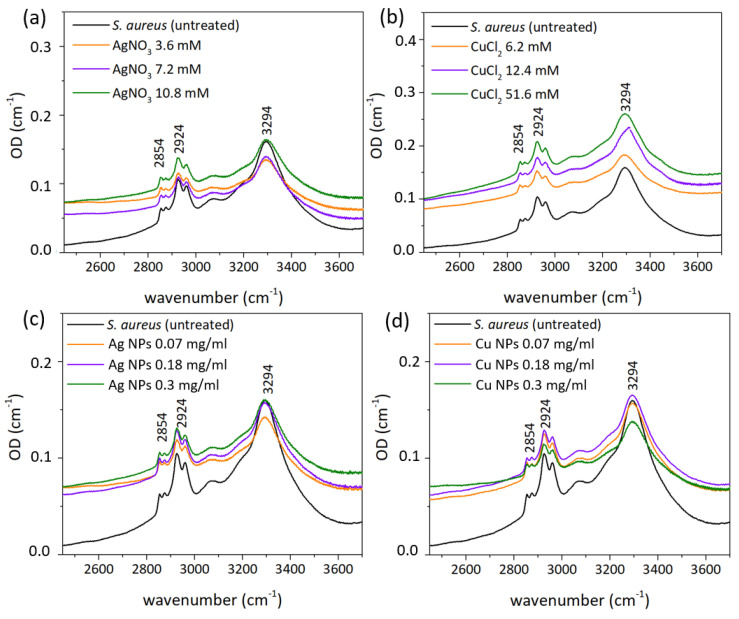
Selected regions (2450–3700 cm^−1^) of FT−IR spectra of *S. aureus* before and after treatment with (**a**) AgNO_3_, (**b**) CuCl_2_, (**c**) Ag NPs, and (**d**) Cu NPs solutions with different concentrations.

Band assignments are presented in Table 1. Spectra (Figure 7, Figure 8 and Figure 9) illustrate the presence of amide A band at ∼3300 cm^−1^ (N–H and O–H stretching vibration of proteins [60,61,62], 2957 cm^−1^ CH_3_ asymmetric stretching of fatty acids [63], 2930 cm^−1^ CH_2_ asymmetric stretching of lipids [64], 2875 cm^−1^ CH_3_ symmetric stretching of proteins [64], 2850 cm^−1^ CH_2_ symmetric stretching of lipids [64], 1737 cm^−1^ C-O stretching of triglycerides and cholesterol esters [64], 1693–1627 cm^−1^ amide I C-O stretching of proteins [65], 1541 cm^−1^ amide II: N–H bending, C–N stretching of proteins [65], 1452 cm^−1^ CH_2_ bending of lipids [66] 1390 cm^−1^ COO− symmetric stretching of amino acid side chains and fatty acids [64], 1300 cm^−1^ amide III band components of proteins [63,64], 1240 cm^−1^ PO_2_ asymmetric stretching of nucleic acids [64,65], 1110 cm^−1^ C-C symmetric stretching of ribose [67], 1082 cm^−1^ PO_2_ symmetric stretching of nucleic acids and phospholipids [62,64,65] 967 cm^−1^ RNA and DNA backbone C-C stretching of nucleic acids [62,67]).

The α-helix type of protein secondary structure is shown in spectra by a band at 1655–1658 cm^−1^ [68], β-sheets exhibit bands at 1637–1623 cm^−1^. Under normal growth conditions of bacterial cells, the maximum of the amide I band (1650–1660 cm^−1^) illustrates the predominance of α-helices among the secondary structure components of bacterial cellular proteins. Bands at 2854 cm^−1^ and 2924 cm^−1^ correspond to symmetrical and asymmetrical stretching vibrations of CH_2_ in lipids. Total band area corresponds to the concentration of the functional group, and its bandwidth reflects the conformational freedom and flexibility, and Lorentz fitting of the bands provided the necessary parameters (Table 2 and Table 3).

Bandwidths of amide I and II bands provide information about conformational freedom of proteins [61,62]. An increase in protein binding leads to the decrease of flexibility and the narrowing of the bandwidth, and in our work the amide II bandwidth narrows only in case of treatment of bacteria with CuCl_2_, which may indicate the binding of the proteins to Cu ions. The considerable broadening of amide bands in all other cases (up to ≈26%) may be caused by the rupture of the membrane. The areas of bands illustrate changes in the concentrations of molecules, to vibrations of which they correspond. Amide I and II bands area increased significantly in all cases except CuCl_2_ treatment (Table 3), which may indicate the increase of protein and polysaccharides concentrations [68].

Membrane lipids are represented in FT-IR spectra as bands at 2854 cm^−1^ and 2924 cm^−1^, which correspond to the symmetric and asymmetric CH_2_ stretching modes [69] and may indicate the disorder of the acyl chains of lipids and the interactions between lipids and membrane proteins. This frequency decrease by 2 cm^−1^ in AgNO_3_ indicates the transition of the membrane lipids from an ordered to a disordered state [70,71,72] and no shift in all other cases. Bands narrow after treatment with CuCl_2_ and Ag NPs (Table 2). Its broadening in all other cases may indicate a higher level of membrane fluidity [72]—the extent of molecular disorder and molecular motion within a lipid bilayer [69]. CH_2_ symmetric and asymmetric stretching bands’ areas decrease after treatment with almost all NPs and metal ion-containing solutions (Table 3), except some samples that were treated with CuCl_2_. This may indicate a lower concentration of saturated lipids [73] and an increase of membrane fluidity.

Amide A band at ≈3300 cm^−1^ demonstrates the increase of bandwidth in all treated samples except CuCl_2_ (Table 2) and overall decrease of area (Table 3).

C–C and PO_2_ symmetric stretching-related bands at ≈1124 cm^−1^ and 1082 cm^−1^ illustrate the vibrations of molecules in nucleic acids [73]. After treatment with metal ion-containing solutions (AgNO_3_, CuCl_2_) the peak at 1082 cm^−1^ disappeared, whereas the peak related to C–C vibrations shifted to a lower frequency (Table 1). After treatment with NPs, the inverse dynamic took place, where the peak at 1124 cm^−1^ disappeared and the PO_2_-related band shifted to a lower frequency.

The band at 966 cm^−1^ is related to RNA and DNA backbone C–C stretching of nucleic acids, and its presence in all spectra without frequency shifts indicates that DNA is in the β-helix form [67] but perhaps with reduced flexibility due to interaction with metal ions. The band at 996 cm^−1^ corresponds to ribose–phosphate main chain vibrations of the ribose skeleton [73] and is present in all spectra, both control and treated. Therefore, according to spectral data, DNA of bacteria are not modified or damaged.

### 3.4. Microbiological Tests

**Figure A1:**
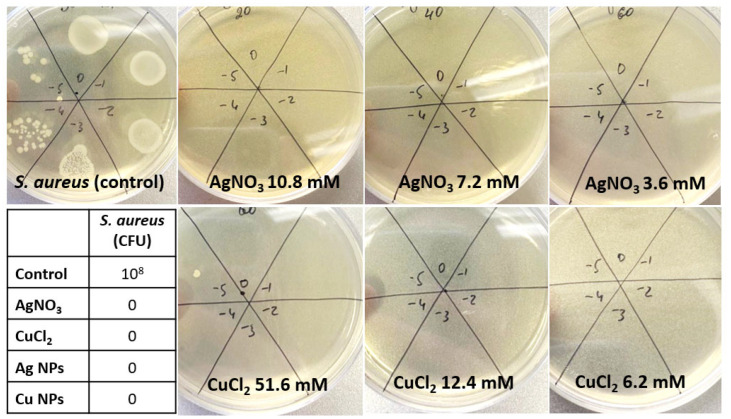
Photograph of *S. aureus* culture, treated with AgNO_3_ and CuCl_2_ solutions with different concentrations and the corresponding table with number of CFU after treatment. Data in the table represent the results for all used concentrations.

Culture of bacteria was implemented as follows: after treatment with NPs and metal-containing solutions, all substrates, including control, were moved to individual sterile testing tubes with saline solution and shaken intensively for 30 min. The resulting suspension was added to the nutrient medium and placed in a thermostat for 24 h at 37 °C. After that, the number of colony forming units (CFUs) was determined and compared with the control samples (Table 4, Figure A1).

Results of *S. aureus* treatment with Ag and Cu NPs, AgNO_3_ and CuCl_2_ solutions with different concentration (Table 4) demonstrate strong antibacterial effect.

In all cases, metal NPs and ion-containing solutions have shown 100% efficiency against *S. aureus.*

The increase of the membrane fluidity, shown in FT-IR spectra, and its subsequent rupture because of Ag and Cu NPs may be related to lipid peroxidation by ROS such as superoxide (O_2_) and hydroxyl radicals (•OH) [74]. Direct contact between NPs and the bacterial membrane is necessary [75,76,77], and while bacteria are known to have a negative charge, it is believed that the positive z-potential of NPs ensure the contact due to electrostatic attraction (Cu NPs in our experiments, Figure 5). Vice versa, electrostatic repulsion between bacterial cell and NPs with negative z-potential is assumed, although in our case negatively charged Ag NPs (Figure 5) still have a strong antibacterial effect. The detected increase of the membrane fluidity and the transition of the membrane lipids from an ordered to a disordered state, as well as NPs precipitation on the cell membranes (Figure 7 and Figure 8), confirms this implication.

It is believed that NPs toxicity on bacterial cells can be explained by NPs penetration in the cell. The largest pores in the bacterial membrane have a mean size of several nm [78,79], and NPs produced by laser ablation have a correspondingly small fraction (Figure 3a). According to TEM data, penetration of NPs and precipitates is observed (Figure 7 and Figure 8).

Cellular uptake of metallic ions leads to the subsequent disruption of DNA functionality and cell death [80,81,82]. It has also been suggested that Cu ions cause damage to phosphorus and sulfur-containing biomolecules, which leads to disruption of intracellular biochemical processes [13,14], which can explain the toxicity of metal salts on bacteria.

Summarizing all of the above, we can propose several individual mechanisms of *S. aureus* death due to Ag NPs, Cu NPs, AgNO_3_ and CuCl_2_.

(1) Ag NPs release ions and ROS, which, due to the close contact of NPs and bacteria in a concentrated (0.3 mg/mL) colloidal solution, and despite negative z-potential, result in lipid peroxidation and cell destruction;
(2) Cu NPs with high positive z-potential are adjacent to bacterial cells via electrostatic attraction and also cause ROS-related lipid peroxidation;
(3) Ag NO_3_ solution forms precipitates on the bacterial cell surface, which lead to the mechanical rupture of the membrane and subsequent penetration of the precipitates in the emerged damaged spots, and to the complete destruction of the membrane and bacterial death;
(4) Cu ions from the CuCl_2_ solution cause damage to phosphorus and sulfur-containing biomolecules, which leads to disruption of intracellular biochemical processes.

## 4. Conclusions

In this work, we studied the bactericidal effect of Ag and Cu NPs in colloidal form and AgNO_3_ and CuCl_2_ solutions with different concentrations on *S. aureus* strain. All samples were characterized by UV-vis spectroscopy, SEM, TEM and EDS. Bacterial inoculation showed considerable antibacterial activity of metal ions-containing salts regardless of their concentration. FT-IR spectroscopy analysis showed the broadening of the amide I and II bands, as well as CH_2_-related peak and its frequency decrease, which indicates the increase of membrane fluidity, leading to bacterial death. TEM analysis confirmed the penetration of Ag NPs and precipitates from AgNO_3_ solution. The main mechanisms of the antibacterial effect were proposed: Ag and Cu NPs release ions and ROS, which result in lipid peroxidation and cell destruction; AgNO_3_ solution forms precipitates on the bacterial cell surface, which lead to the mechanical rupture of the membrane and subsequent possible penetration of the precipitates in the emerged damaged spots, and to complete destruction of the membrane and bacterial death; Cu ions from the CuCl_2_ solution cause damage to phosphorus and sulfur-containing biomolecules, which leads to disruption of intracellular biochemical processes.

## Figures and Tables

**Table 1:** IR band assignments of bacteria (treated with antibacterial agents and control).

Functional Groups	Frequency (cm^−1^)				
	Control	AgNO_3_-Treated	Ag NP-Treated	CuCl_2_-Treated	Cu NP-Treated
Amide A	3294	3294	3294	3294	3294
CH_2_ asym str	2924	2922	2925	2925	2925
CH_2_ sym str	2854	2852	2854	2854	2852
Amide I	1656	1656	1655	1655	1655
Amide II	1544	1541	1541	1543	1541
C-C	1124	1104	-	1106	-
PO_2_	1082	-	1082	-	1085
ribose skeleton	997	997	997	997	997

**Table 2:** Differences in bandwidths of peaks between control and treated bacteria.

Functional Groups	Bandwidth (cm^−1^)		%Change		%Change		%Change		%Change
	Control	AgNO_3_-Treated		Ag NP-Treated		CuCl_2_-Treated		Cu NP-Treated	
Amide I	53.5 ± 2.35	61.39 ± 1.83	+14.75	63.28 ± 1.67	+18.28	55.69 ± 1.28	+4.09	64.17 ± 1.64	+19.94
Amide II	57.19 ± 2.74	64.59 ± 1.83	+12.94	68.54 ± 1.34	+19.85	52.71 ± 1.53	−7.83	70.30 ± 1.45	+22.92
CH_2_ sym str	8.82 ± 2.04	10.18 ± 1.81	+15.4	8.82 ± 1.79	0	11.54 ± 2.63	+30.84	8.56 ± 1.75	−2.95
CH_2_ asym str	33.5 ± 1.75	33.76 ± 1.43	+0.8	33.81 ± 1.64	+0.93	30.54 ± 2.22	−8.84	34.46 ± 1.52	+2.09
Amide A	156.24 ± 2.42	164.21 ± 2.33	+5.11	157.32 ± 1.96	+0.69	147.59 ± 2.2	−5.54	173.07 ± 2.19	+10.77

**Table 3:** Differences in area of peaks between control and treated bacteria.

Functional Groups	Area (a.u.)		%Change		%Change		%Change		%Change
	Control	AgNO_3_-Treated		Ag NP-Treated		CuCl_2_-Treated		Cu NP-Treated	
Amide I	21.51 ± 0.36	17.48 ± 0.17	−18.74	17.12 ± 0.15	−20.41	22.43 ± 0.4	+4.28	14.47 ± 0.12	−32.73
Amide II	11.6 ± 0.38	10.57 ± 0.1	−8.88	10.66 ± 0.17	−8.1	10.95 ± 0.4	−5.6	9.48 ± 0.14	−18.28
CH_2_ sym str	0.18 ± 0.04	0.18 ± 0.04	0	0.13 ± 0.03	−27.8	0.24 ± 0.06	+33.34	0.1 ± 0.02	−44.4
CH_2_ asym str	2.51 ± 0.18	1.96 ± 0.1	−21.91	1.63 ± 0.1	−35.06	2.04 ± 0.24	+18.73	1.42 ± 0.08	−43.43
Amide A	29.35 ± 0.71	20.68 ± 0.48	−29.54	17.92 ± 0.33	−38.94	25.52 ± 0.58	−13.05	18.01 ± 0.35	−38.64

**Table 4:** Number of *S. aureus* CFU after treatment with Ag, Cu NPs, AgNO_3_ and CuCl_2_ solutions with different concentrations. Data in the table represent the results for all used concentrations.

	*S. aureus* (CFU)
control	108
AgNO3	0
CuCl2	0
Ag NPs	0
Cu NPs	0

## Data Availability

The data presented in this study are available on request from the corresponding author.
